# The Effect of Glaucoma Medication on Choroidal Thickness Measured with Enhanced Depth-Imaging Optical Coherence Tomography

**Published:** 2019

**Authors:** Serife BAYRAKTAR, Zafer CEBECI, Belgin IZGI, Kamber KASALI

**Affiliations:** 1 Department of Ophthalmology, Istanbul Faculty of Medicine, Istanbul University, Istanbul, Turkey; 2 Department of Bioistatistics, Ataturk University, Erzurum, Turkey

**Keywords:** Primary Open-Angle Glaucoma, Glaucoma Medication, Choroidal Thickness, Enhanced Depth-imaging, Optical coherence Tomography

## Abstract

The aim of this study was to examine the effect of the glaucoma medication on Choroidal Thickness (CT) in those with Primary Open-Angle Glaucoma (POAG) and normal cases. This prospective study included 27 patients with newly diagnosed POAG (group 1; 49 eyes), undergoing glaucoma treatment, and 30 patients, whose treatment was terminated due to misdiagnosis (group 2; 57 eyes). Choroidal thickness was measured using Enhanced Depth Imaging (EDI) with Spectral Domain Optical Coherence Tomography (SD-OCT) at the first visit and almost one month later. In group 1, the mean Sub-Foveal CT (SFCT) was 301 ± 91 µm, the mean CT was 264 ± 87 µm at the nasal point, 1 mm to the fovea, and 271 ± 84 µm at the temporal point, 1 mm to the fovea. The second measurements were obtained as 39 ± 8.5 days after treatment began; the SFCT was 319 ± 85 µm (P = 0.0017), the nasal 1 mm CT was 275 ± 88 µm (P = 0.162), and the temporal 1mm CT was 291 ± 80 µm (P = 0.007). In group 2, the mean SFCT was 292 ± 100 µm, the nasal 1 mm CT was 254 ± 97 µm, and the temporal 1 mm CT was 261 ± 97 µm. The second measurements were obtained 37.5 ± 5.5 days after the treatment ended; the SFCT was 295 ± 107 µm (P = 0.212), the nasal 1 mm CT was 262 ± 104 µm (P = 0.709), and the temporal 1 mm CT was 266 ± 104 µm (P = 0.792). Glaucoma medication affects the CT as a marker for choroidal blood flow in patients with glaucoma. Further studies with larger sample sizes are required to examine each glaucoma medication subgroup.

## INTRODUCTION

Glaucoma is a significant cause of blindness in many countries, irrespective of their development status. Typical morphologic changes in the Optic Nerve Head (ONH) occur after damage to the ganglion cells and their axons, and this is known as Glaucomatous Optic Neuropathy (GON) [1-4]. Progressive changes in the optic disc, retinal nerve fiber layer, and ganglion cell-inner plexiform layer, peripheral and central visual fields are perceived in GON. Intraocular Pressure (IOP) is majorly accepted as a significant risk factor in glaucoma, and although the progression of vision and visual field loss can be slowed or stopped by IOP reduction, in Normal-Tension Glaucoma (NTG), it may persist regardless of whether IOP is medically or surgically controlled. This indicates that mechanisms beyond IOP, such as worsening choroidal blood flow or ocular ischemia, may be instrumental in the deterioration of vision loss [5-10]. There have been conflicting results in studies that evaluated Choroidal Thickness (CT) in patients with glaucoma as a sign of choroidal blood flow, both in histologic and imaging studies [11-13]. Histologic studies can be influenced by long-term perfusion loss to the eye and by the sample preparation method used. Imaging is a prerequisite for in vivo investigations of the choroid in glaucoma. Enhanced Depth Imaging (EDI) technique of Spectral Domain Optical Coherence Tomography (SD-OCT) instruments has resulted in improved choroid images [14-16]. Recently, a number of studies have compared CT between patients with Primary Open-Angle Glaucoma (POAG) and controls, yet the results have not been conclusive. Macular or peripapillary choroidal thinning in patients with POAG or NTG was reported in some cases yet most previous studies showed no significant differences between patients with glaucoma and healthy individuals or among those, who are thought to have glaucoma [17-25]. Accordingly, further studies examining this issue are merited. The aim of glaucoma medication is to lower IOP, which is a major factor in the pathogenesis of this condition. Future treatment methodologies may include vaso-protective drugs, which influence blood flow or neuroprotective medication. Choroidal thickness, a marker of choroidal flow, can be affected by the type of glaucoma medication administered [5-10]. Thus, this study evaluated the effect of glaucoma medication on CT, using SD-OCT, with the EDI technique.

## METHODS

This prospective study consisted of 27 patients with newly diagnosed POAG (group 1; 49 eyes; 15 females, 12 males), and 30 patients (group 2; 57 eyes; 19 females, 11 males), whose glaucoma treatment was terminated due to the misdiagnosis of glaucoma (normal subjects). In group 1, Sub-Foveal Choroidal Thickness (SFCT) was measured before anti-glaucomatous treatment and approximately one month after the treatment began. In group 2, the SFCT was measured during anti-glaucomatous treatment and approximately one month after termination of the treatment in order to wait for the wash-out period. The study protocol was approved by the Ethics Committee of Istanbul Faculty of Medicine and all of the patients provided an informed consent. All researches were conducted in accordance with the Declaration of Helsinki.

The exclusion criteria for both groups were diabetes mellitus, systemic arterial hypertension, use of systemic corticosteroids, macula diseases, ocular surgery or trauma, inflammation, non-glaucomatous optic neuropathy, or corneal opacities, and smoking. Inclusion criteria were having a Corrected Distance Visual Acuity (CDVA) of≥20/40, within -5 to +3 diopters (D) of refractive errors, and ± 3D cylindrical correction, with a clear cornea and clear ocular media as well as diagnosis of POAG in Group 1. The inclusion criteria for Group 2 were misdiagnosis of POAG, being under treatment of anti-glaucomatous medication, having a CDVA of 20/20, a normal-appearing optic disc, normal Retinal Nerve Fiber Layer (RNFL) thickness measurement, a normal visual field, no ocular pathology or ocular trauma, and no familial history of glaucoma.

All patients underwent detailed ophthalmologic assessments, including quantification of CDVA, slit-lamp biomicroscopy, Goldmann applanation tonometry, gonioscopy, indirect ophthalmoscopy, RNFL thickness using SPECTRALIS® OCT (Heidelberg Engineering, Heidelberg, Germany), and achromatic automated perimetry, using the 30-2 Swedish Interactive Threshold Algorithm (SITA) standard program (Humphrey Visual Field Analyzer; Carl Zeiss-Meditec, Inc., Dublin, CA, USA). The POAG was defined as the presence of an abnormal glaucomatous optic disc, an abnormal visual field correlated with the decrease in RNFL thickness, an IOP > 21 mm Hg (without medical treatment), and an open-angle as determined by gonioscopy.

**Figure 1:**
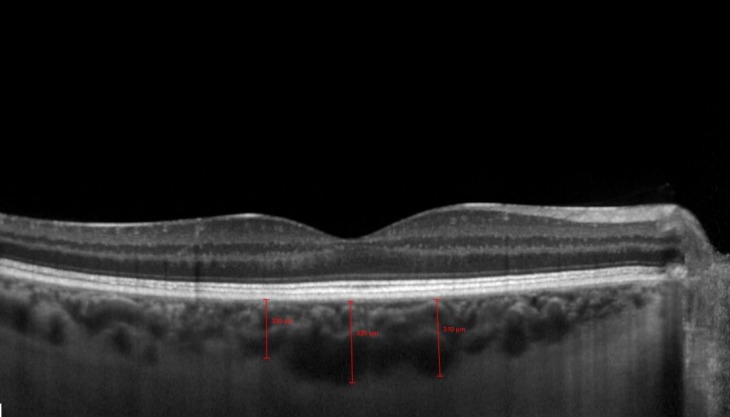
The Enhanced Depth Imaging (EDI) Photograph using Spectral Domain Optical Coherence Tomography (SD-OCT) showing the Measurement of Choroidal Thickness.

Each patient was examined using SPECTRALIS HRA-OCT in the EDI mode. The choroid was imaged by positioning the SD-OCT device sufficiently close to the eye to obtain a clear image. Each image was averaged from 100 scans using the automated averaging and eye-tracking features provided by the SPECTRALIS HRA-OCT. The SFCT was measured vertically from the outer border of the hyper-reflective band corresponding to the retina pigment epithelium to the inner surface of the sclera, using the caliper tools of the software (Heidelberg Eye Explorer Version 1.6.4.0; Heidelberg Engineering). Choroidal thickness was measured at three points: At the SFCT and at 1 mm nasal and 1 mm temporal to the fovea. A single experienced technician took all the measurements. One of the measurements is shown in Figure 1. All EDI-OCT measurements were made in the morning in order obviate the effect of diurnal variation in CT.

The statistical analyses performed using SPSS 20.0 (SPSS, Inc., Chicago, IL, USA). The Shapiro Wilk (SW) test was applied to study normality of distribution. The Wilcoxon test was used to verify statistically significant differences between the two groups. Regarding continuous variables, for normally distributed variables, independent samples t-test was used and for non-normally distributed variables, the Mann-Whitney U test was used. The Chi-square test was used for categorical variables. For all tests, P values of < 0.005 were accepted as statistically significant.

## RESULTS

In this prospective study, the researchers examined 106 eyes of 57 patients (34 females and 23 males). The mean ± Standard Deviation (SD) of age was 55.6 ± 14.7 years. The patients’ clinical characteristics are summarized in Table 1. 

In group 1 (49 eyes; 27 patients with newly diagnosed POAG), the mean ± SD of age of the participants was 55.6 ± 12 years. The mean spherical equivalent was -0.11 ± 1.8 D. At the time of diagnosis, the mean IOP was 23.5 ± 1.5 millimeters of mercury (mm Hg) and the mean average RNFL thickness was 92 ± 18 micrometers (µm). The mean ± SD of SFCT was 301 ± 91 µm; at the nasal point 1 mm to the fovea, the mean ± SD of CT was 264 ± 87 µm, and at the temporal point 1 mm to the fovea, the mean ± SD of CT was 271 ± 84 µm. 

The glaucoma medications included prostaglandin analogs in 29 eyes, carbonic anhydrase inhibitors + timolol fixed-combinations in five eyes, brimonidine tartrate in two eyes, brimonidine tartrate + timolol fixed-combination in nine eyes, and bimatoprost + timolol fixed-combination in four eyes. The details of the drugs are shown in Table 2. The second IOP and CT measurements were 39 ± 8.5 days after the treatment began. The second mean ± SD of IOP was 15.3 ± 2.4 mm Hg (P < 0.001). The second mean ± SD of SFCT was 319 ± 85 µm (P = 0.0017), the mean ± SD of nasal 1 mm CT was 275 ± 88 µm (P = 0.162), and the mean ± SD of temporal 1 mm CT was 291 ± 80 µm (P = 0.007). The comparison of CT in group 1 before and after treatment is presented in Table 3.

In group 2 (57 eyes and 30 patients), glaucoma treatment was terminated due to misdiagnosis and no other ophthalmologic pathologies were found. The mean ± SD of age of the participants was 55.6 ± 17 years. The mean ± SD of spherical equivalent was 0.4 ± 2.7 D. The mean ± SD of IOP at the time of diagnosis was 15 ± 3.2 mm Hg and the mean ± SD of average RNFL thickness was 102 ± 9 µm. The mean ± SD of SFCT was 292 ± 100 µm; at the nasal point 1 mm to the fovea, the mean ± SD of CT was 254 ± 97 µm and at the temporal point 1 mm to the fovea, the mean ± SD of CT was 261 ± 97 µm. The following glaucoma medications were terminated: Prostaglandin analogs in 32 eyes, carbonic anhydrase inhibitors + timolol fixed-combinations in eight eyes, brimonidine tartrate in three eyes, brimonidine tartrate + timolol fixed-combination in two eyes, prostaglandin analogs + timolol fixed-combination in four eyes, carbonic anhydrase inhibitors in two eyes, and timolol maleate in six eyes. The details of these drugs are shown in Table 2. The second IOP and CT measurements were obtained as 38 ± 5.5 days after treatment ended. The second mean ± SD of IOP was 15.5 ± 2.5 mmHg (P > 0.001). The second mean ± SD of SFCT was 295 ± 107 µm (P=0.212), the mean ± SD of nasal 1 mm CT was 262 ± 104 µm (P=0.709), and the mean ± SD of temporal 1 mm CT was 266 ± 104 µm (P=0.792). The comparison of CT in group 2 of those under treatment and without treatment is shown in Table 4.

**Table 1:** Clinical Characteristics in Study Participants of both Groups (n = 106)

	Group 1, (n=49)	Group 2, (n=57)	P
Age(y)	56 ± 12	56 ± 17	0.999
Spherical Equivalent, D	-0.11 ± 1.8	0.42 ± 2.74	0.89
Central Corneal Thickness, µm	537 ± 37	552 ± 37	0.98
RNFL Average Thickness, µm	92 ± 18	102 ± 9	0.007*

Data are presented as mean ± SD. SD: standard deviation; n: number; y: year; D: diopter; µm: micrometer; RNFL: retinal nerve fiber layer.

Note: Independent samples t test and Mann Whitney U test applied for analysis.

* : P value less than 0.05.

**Table 2:** Details of the anti-glaucoma drugs started or terminated in both groups

Brand name	Active ingredient	Group1 (n=49)	Group 2 (n=57)
Lumigan® ^1^	Bimatoprost 0.01%	20	2
Xalatan®^2^	Latanoprost 0.005%	9	27
Travatan®^3^	Travoprost 0.004%	0	3
Alphagan-P®^1^	Brimonidine tartrate 0.15%	2	3
Combigan®^1^	Timolol maleate 0.5%+ brimonidine tartrate 0.2%	9	2
Azarga®^3^	Brinzolamide 1% + timolol maleate 0.5%	3	2
Cosopt®^4^	Dorzolamide 2% + Timolol maleate 0.5%	2	6
Ganfort®^1^	Bimatoprost 0.001% + Timolol maleate 0.5%	4	0
Xalacom®^2^	Latanoprost 0.005% + Timolol maleate 0.5%	0	2
Duotrav®^3^	Travoprost 0.004%+ Timolol maleate 0.5%	0	2
Azopt®^3^	Brinzolamide 1%	0	2
Timoptic XE®^4^	Timolol maleate 0.5%	0	6

1 Allergan, Inc., Irvine, CA, USA;

2 Pfizer, Inc., New York, NY, USA;

3 Alcon laboratories, Inc., Fort Worth, TX, USA;

4 Merck & Co., Inc, Blue Bell, PA

**Table 3:** Comparison of choroidal thicknesses in Group 1 before and after the treatment

	Before treatment	After treatment	P
Choroidal thicknesses(µm ± SD)			
Subfoveal	301 ± 91	319 ± 85	0.0017*
Nasal 1mm to fovea	264 ± 87	275 ± 88	0.162
Temporal 1 mm to fovea	271 ± 84	291 ± 80	0.007*

SD: standard deviation; µm: micrometer; mm:milimeter. Note: Wilcoxon test applied for analysis.

* : P value less than 0.05.

## DISCUSSION

This study aimed at evaluating the effect of glaucoma medication on CT using SD-OCT with the EDI technique. The results indicated that the mean sub-foveal CT was significantly higher after topical anti-glaucomatous medication treatment in patients, who were newly diagnosed as POAG. However, there was no significant difference in normal subjects. 

Numerous studies have investigated choroid in glaucoma by measuring CT thickness, using histopathologic or *in vivo* imaging techniques. Histologic studies have shown divergent CT results in post-mortem glaucoma studies [12, 13]. Yin et al. found a decrease in CT, which they attributed to the coexistence of decreased vessel density and the reduced patency of choroidal vessels. In contrast, Spraul et al. found an increase in both calibers of the largest choroidal arteries and in the CT [12, 13]. The disparity between these studies may be related to the methods used.

**Table 4:** Comparison of choroidal thicknesses in Group 2 under treatment and without treatment

	Under treatment	Without treatment	P
Choroidal thicknesses(µm ± SD)			
Subfoveal	292 ± 100	295 ± 107	0.212
Nasal 1mm to fovea	254 ± 97	262 ± 104	0.709
Temporal 1 mm to fovea	261 ± 97	266 ± 104	0.792

SD: standard deviation; µm: micrometer; mm:milimeter. Note: Wilcoxon test applied for analysis.

Newer techniques that can measure CT in vivo, such as SD-OCT, are more likely to be accurate. The SD-OCT coupled with a technique called EDI, which was described by Margolis and Spaide, has the ability to image beyond the retinal pigment epithelium [14, 15]. These studies showed that CT decreased with age, and in eyes with a longer axial length, the choroid was thinner. Thus, many studies have investigated CT within the macula because the choroid is thickest under the fovea in glaucomatous eyes. In many published studies, no meaningful differences were found between the macular CT of glaucomatous eyes in comparison with those of controls [20-23, 26]. Mwanza et al. studied one eye in 38 normal patients, 20 with NTG, and 56 with POAG and they could not find any relationship between glaucoma and average macular CT [20]. In their study, patients were also grouped, according to the degree of visual field change; nevertheless, no meaningful difference in CT was determined between the groups. In another study by the same group, the affected eyes of patients with unilateral advanced glaucoma were compared with their unaffected eyes [23]. In this way, variables, such as age and comorbidities, were eliminated, yet still no differences were found. Rhew et al. measured SFCT in 32 patients with NTG, whose results were compared with 35 healthy controls and they found no meaningful difference [22]. Jonas et al. measured CT in 71 patients with glaucoma and 228 normal individuals, and their findings revealed no significant differences [26]. Toprak et al. assessed CT and vessel diameter in patients with POAG using SD-OCT EDI mode with age-based analysis and compared them with healthy subjects [27]. They found no significant difference in CT and vessel caliber between patients with POAG and healthy controls. Wang et al. examined the CT of patients with POAG using EDI-OCT and compared them with healthy subjects [28]. They also performed a meta-analysis about this among Chinese patients. They suggested that POAG was not significantly associated with a marked thinning or thickening of the choroid based on EDI-OCT measurements. The findings of the above studies are in contrast with the findings of the current study. The reason for this difference could be due to the differences in the race of the subjects, the course of the disease, the type of drugs used and other causes that are still unknown and should be investigated in future studies in details. 

In contrast, Hirooka et al. suggested that CT was focally diminished in patients with glaucoma [19]. The authors studied 62 normal eyes and 45 eyes with NTG and investigated the difference. They found a meaningful choroidal thinning in the nasal fovea of NTG patients. Roberts et al. compared 92 controls with glaucoma patients, including focal (n = 34), diffuse (n = 35), and sclerotic (n = 34) optic disc damage [18]. They only found that the choroid was thinner in eyes with sclerotic disc damage. Ersoz et al. investigated the difference of CT and factors influencing CT between POAG patients and the control group by using SD-OCT EDI mode [29]. They found that CT was thinner in the POAG group. However, this was suggested to arise from the difference in IOP and pulse rate between the groups. The findings reported in these studies revealed that open-angle glaucoma is not associated with the thickening of the choroid in the foveal or para-foveal region, which differs from our results that could be contributed to reasons mentioned earlier. 

The researchers aimed to study the impact of glaucoma medication on CT as a sign of choroidal flow and ocular perfusion. In POAG, the researchers found a significant difference in CT under anti-glaucomatous treatment. In most cases, glaucoma medication is used to lower IOP to protect against the deleterious effect on neuro-retinal tissues. Many existing medications interact with the vasculature and change ocular blood flow. Many previous studies have examined the effect of subclasses of medications on ONH and retinal, choroidal, and retro-bulbar circulation [30-43].

By blocking carbonic anhydrase type II, the topical Carbonic Anhydrase Inhibitor (CAI), dorzolamide, significantly lowers IOP. The efficacy of CAI, as a vasodilator, has been demonstrated by many studies [30]. Using color Doppler imaging, Martinez et al. evaluated the effect of dorzolamide on ocular blood flow in normal and glaucomatous eyes. They showed higher end-diastolic velocities in the ophthalmic and central retinal artery and also increased peak-systolic velocity in the central retinal artery in glaucomatous eyes [31]. Dorzolamide was also found to accelerate the retinal arteriovenous flow rate of fluorescein dye without affecting flow speeds in any retrobulbar vessels in patients with NTG [32]. Recently, it has been reported that another CAI brinzolamide has a better affinity. In rabbit studies it was shown that it is a good vasodilatation effect and thus results a higher blood flow in the ONH [33]. However, there are no data concerning the effects of dorzolamide and brinzolamide on choroidal flow or CT.

Different studies evaluated the use of topical β-blockers as a local anti-glaucomatous medication. Using laser Doppler velocimetry, one study found that treatment with timolol maleate (0.5%) resulted in a substantial difference in retinal blood flow in healthy controls [34]. Conflictingly, Yoshida et al. found no difference in blood flow after the same treatment [35]. It might be considered, therefore, that these β-blocker studies were unpersuasive regarding drug penetration and their effects on ocular blood flow [36]. 

Another group of drugs that might be considered for their possible vasoactive effects are alpha-adrenergic agonists. Using laser Doppler flow metry, Carlsson et al. showed that topical brimonidine has no retinal blood flow alteration [37]. The literature includes studies that found that brimonidine tartrate had no impact on ocular hemodynamics, which may be because of the very slight penetration to the posterior pole [38].

The mechanism of action of prostaglandin analogs, through increasing uveoscleral outflow, subsequently reduces IOP. Using scanning laser Doppler flowmetry, two hours and 24 hours after administration of latanoprost drops (0.005%) in healthy volunteers, Seong et al. reported no meaningful difference in the ONH and peripapillary retinal blood flow [39]. In contrast, other studies with pulsatile Ocular Blood Flow (pOBF) measured a positive effect on ONH blood flow [40, 41]. Vetrugno et al. used pOBF to study the contrast in the effects of bimatoprost and timolol on IOP and choroidal blood flow in POAG patients and found that bimatoprost increased blood flow [42]. Koz et al. used color Doppler ultrasound to investigate the effect of three prostaglandin analogs (latanoprost, travoprost, and bimatoprost) on retro-bulbar blood flow velocity in previously untreated POAG or Ocular Hypertension (OHT) patients [43]. Their results suggested that the three prostaglandin analogs meaningfully reduced IOP and increased ocular perfusion pressure in all patients. Topical travoprost and latanoprost significantly reduced the resistive index of the central retinal artery and ophthalmic artery, respectively. However, the authors observed that topical bimatoprost did not influence ocular hemodynamics [43].

In summary, all these studies showed that the effect of glaucoma medications on ocular, retinal, or choroidal blood flow is still controversial. Moreover, there are no data on the effect of these medications on CT. The current study aimed at identifying the effect of anti-glaucomatous medication on CT using SD-OCT in EDI mode. In group one, the patients were newly diagnosed as having POAG and the mean sub-foveal CT was significantly higher after a treatment period of approximately 40 days, as shown in Table 3. In group 2, the patients were misdiagnosed as having glaucoma, thus their treatments were terminated. Therefore, it could be suggested that group 2 was a control group. There was no significant difference in the mean CT in group 2 after the wash-out period, as shown in Table 4.

The present study had many limitations. First, the sample size of the two groups was relatively small. Second, the type of glaucoma medication that was administered was not the same for each patient or each group. It would be better to examine each subgroup of anti-glaucomatous medication in the same patients because there can be differences based on the type of medications used. Third, this study only measured CT in the subfoveal region and the parafoveal area, and did not measure peripapillary CT, which is suggested to have a greater association with glaucoma. Fourth, the measurements were taken by a single technician with a manual caliper. In conclusion, the current study showed that glaucoma medication affects CT as a marker for choroidal blood flow in patients with glaucoma. Drugs that influence blood flow, thereby act as vasoprotective and neuroprotective medications, and might be options to investigate in the future. Thus, additional studies with larger sample sizes are needed to examine each glaucoma medication subgroup and should include a longer follow-up period.
